# Percepciones de enfermeras sobre la integración de la experiencia y los resultados de salud reportados por pacientes en la mejora de los cuidados: Estudio cualitativo multicéntrico

**DOI:** 10.23938/ASSN.1169

**Published:** 2026-08-18

**Authors:** Cayetana Ruiz-Zaldibar, Pilar Montes-Muñoz, Mayte Ruano-Sanz, Desiree Cidoncha-López, Sandra Crespo-Hernández, María de la Loma Segarra-Cañamares

**Affiliations:** 1 Universidad Camilo José Cela Facultad HM de Ciencias de la Salud Villanueva de la Cañada Madrid España; 2 Instituto de Investigación Sanitaria HM Hospitales Madrid España

**Keywords:** Medidas de Resultados Reportados por el Paciente, Medidas de Experiencia Reportadas por el Paciente, Atención de Enfermería, Investigación Cualitativa, Calidad de la Atención de salud, Patient Reported Outcome Measures, Patient Experience, Nursing Care, Qualitative Research, Quality of Health Care

## Abstract

**Fundamento:**

Este estudio explora la percepción de enfermeras de distintas áreas sobre la utilidad de PREMs y PROMs para identificar oportunidades de mejora de los cuidados y orientar futuras iniciativas de calidad centradas en la experiencia (PREMs) y los resultados de salud (PROMs) reportados por los pacientes.

**Material y métodos:**

Estudio cualitativo interpretativo, multicéntrico y exploratorio, realizado entre noviembre de 2025 y febrero de 2026 mediante cinco grupos focales. Participaron 32 enfermeras de hospitalización, cuidados intensivos de adultos y neonatal, urgencias, quirófano y oncología de cuatro hospitales HM de Madrid. La selección fue intencional. Los datos se recogieron con una guía semiestructurada y se analizaron mediante análisis temático inductivo.

**Resultados:**

Se identificaron seis temas: 1) comunicación, vínculo terapéutico y trato humano como principales determinantes de la experiencia del paciente; 2) cuidado integral, especialmente confort, seguridad clínica y alivio del dolor; 3) información clínica y educación sanitaria como elementos clave para reducir incertidumbre y favorecer continuidad asistencial; 4) valoración positiva de PREMs y PROMs, aunque con uso todavía poco sistemático; 5) limitaciones organizativas, sobrecarga y falta de tiempo como barreras principales; y 6) propuestas relacionadas con la innovación, humanización, tecnología y práctica avanzada orientadas al paciente.

**Conclusión:**

Las enfermeras consideran relevantes la comunicación, el cuidado integral y la educación sanitaria en la experiencia y los resultados percibidos por los pacientes. Asimismo, evidencian una brecha entre el valor reconocido de los PREMs y PROMs y su utilización sistemática en la práctica clínica, condicionada por barreras organizativas y estructurales.

## Abstract (en)

**Background:**

Patient-reported experience measures (PREMs) and patient-reported outcome measures (PROMs) are increasingly recognized as valuable tools for evaluating healthcare quality. This study aims to explore nurses’ perceptions across diverse clinical settings regarding the usefulness of PREMs and PROMs for identifying opportunities to improve care and informing quality improvement initiatives.

**Materials and methods:**

Interpretive, exploratory, multicenter qualitative study conducted between November 2025 and February 2026. Five focus groups were held with 32 nurses recruited through purposive sampling from inpatient wards, adult and neonatal intensive care units, emergency departments, operating rooms, and oncology units across four HM hospitals in Madrid. Data were collected using a semi-structured interview guide and analyzed through inductive thematic analysis.

**Results:**

Six themes emerged: 1) communication, therapeutic relationship, and humanized care as key determinants of patient experience; 2) holistic care, particularly comfort, patient safety, and pain management; 3) clinical information and patient education to reduce uncertainty and improve continuity of care; 4) positive perceptions of PREMs and PROMs despite their limited systematic implementation; 5) organizational constraints, workload, and time pressure as major barriers; and 6) opportunities for innovation through humanization strategies, technology integration, and advanced nursing practice.

**Conclusions:**

Nurses perceive PREMs and PROMs as valuable resources for improving patient-centered case and supporting quality improvement. However, their integration into routine clinical practice remains limited by organizational and structural barriers. Addressing these challenges may facilitate the systematic use of patient-reported measures to enhance care quality and patient outcomes.

## INTRODUCCIÓN

En los últimos años, la evaluación de la calidad asistencial ha evolucionado desde un enfoque centrado exclusivamente en los resultados clínicos hacia una visión más integral que incorpora la percepción y experiencia de los propios pacientes. En este contexto, surgen los *Patient-Reported Experience Measures* (PREMs) y los *Patient-Reported Outcome Measures* (PROMs) como herramientas fundamentales para valorar la calidad de los cuidados desde la perspectiva del usuario^1^^-^^3^.

Los PREMs hacen referencia a la experiencia comunicada por el paciente en relación con el proceso asistencial, es decir, cómo percibe la calidad del trato recibido, la comunicación, la información, la coordinación entre profesionales y su participación en la toma de decisiones^4^. Por su parte, los PROMs valoran los resultados de salud comunicados directamente por el paciente, como el dolor, la funcionalidad, el bienestar emocional o la calidad de vida tras una intervención^5^^,^^6^. A diferencia de otros indicadores habitualmente utilizados para evaluar la calidad asistencial (indicadores clínicos tradicionales, medidas registradas por profesionales, cuestionarios de satisfacción), los PREMs y PROMs permiten una evaluación centrada en la persona, luego responden a constructos diferentes y complementarios.

La participación en la Encuesta Internacional de Indicadores de Salud Referidos por los Pacientes (PaRIS) promovida por la Organización para la Cooperación y el Desarrollo Económicos (OCDE), entre otras iniciativas, ha impulsado en España el interés por incorporar medidas reportadas por los pacientes en los últimos años. Estas acciones reflejan la creciente importancia de integrar la experiencia y los resultados informados por los pacientes en la evaluación y mejora de la calidad de los procesos asistenciales y en el desarrollo de modelos de atención centrados en la persona^7^. Los PREMs y PROMs se han identificado como herramientas relevantes para incorporar la perspectiva del paciente en la evaluación de la calidad asistencial junto con otras estrategias dirigidas a fortalecer la coordinación entre niveles asistenciales, la educación sanitaria y la participación activa de los pacientes en su cuidado^8^.

En la práctica enfermera resultan esenciales para evaluar la efectividad real de los cuidados y su impacto en la experiencia y bienestar del paciente^9^^,^^10^. Comprender cómo los pacientes viven y valoran su proceso asistencial permite identificar fortalezas, áreas de mejora y factores que influyen en su recuperación y satisfacción. No obstante, para que estos datos sean realmente útiles, es necesario integrar la perspectiva del personal de enfermería, quien posee un conocimiento práctico y contextual de las dinámicas clínicas y organizativas y puede aportar información valiosa para comprender cómo integrar los PREMs y PROMs en la práctica asistencial y orientar iniciativas de mejora de la calidad del proceso asistencial^11^. Las enfermeras ocupan una posición especialmente relevante para interpretar la experiencia y los resultados reportados por los pacientes, ya que mantienen una interacción continuada a lo largo del proceso asistencial y participan directamente en la planificación, ejecución y evaluación de los cuidados, aportando información que explica los resultados y orienta estrategias factibles de mejora^12^. Además, su participación en la interpretación y aplicación de estas medidas favorece la implicación en la cultura de calidad y seguridad del paciente, al tiempo que refuerza el modelo de atención centrada en la persona^13^.

Por tanto, explorar las percepciones de las enfermeras sobre los PREMs y PROMs puede contribuir a comprender mejor cómo integrar estas medidas en la práctica asistencial y generar conocimiento útil para futuras iniciativas de evaluación y mejora de la calidad centradas en el paciente^14^.

Aunque los PREMs y PROMs se utilizan cada vez más para incorporar la perspectiva del paciente en la evaluación de la calidad asistencial, sigue siendo limitada la evidencia sobre cómo los profesionales de enfermería interpretan esta información. Por tanto, el objetivo de este estudio es explorar las percepciones de enfermeras de distintas áreas asistenciales sobre la utilidad de los PREMs y PROMs para 1) identificar los elementos del cuidado que más influyen en la experiencia del paciente, 2) analizar los indicadores de salud más representativos de los resultados reportados por el paciente, y 3) detectar áreas de mejora organizativa.

## MATERIAL Y MÉTODOS

### Diseño del estudio

Se realizó un estudio cualitativo interpretativo con un enfoque exploratorio basado en la técnica de grupos focales durante noviembre de 2025 a febrero de 2026. El objetivo general fue explorar la percepción de enfermeras de distintas áreas sobre PREMs y PROMs para identificar potenciales mejoras en los procesos asistenciales.

El estudio adoptó un diseño multicéntrico, incorporando enfermeras de unidades de hospitalización, de cuidados intensivos de adultos y neonatales, urgencias, quirófano y oncología de los hospitales HM Madrid, HM Sanchinarro, HM Puerta del Sur y HM Torrelodones (Comunidad de Madrid, España). Esta diversidad permitió obtener una visión amplia de la experiencia del paciente en distintos niveles de complejidad clínica y tipos de procesos asistenciales, favoreciendo la riqueza analítica y la transferencia de resultados.

### Participantes

La selección de participantes se realizó mediante muestreo intencional, con los siguientes criterios de inclusión: enfermeras con contrato estable en la unidad, desempeño continuado durante al menos seis meses, y disponibilidad para participar. Estos criterios permitieron seleccionar profesionales con experiencia asistencial continuada y conocimiento directo de las dinámicas organizativas y de cuidado, favoreciendo la obtención de información relevante para los objetivos del estudio. La participación fue voluntaria y se obtuvo consentimiento informado.

Las participantes fueron contactadas mediante invitación institucional canalizada a través de las supervisoras de unidad, quienes informaron del estudio y facilitaron la participación voluntaria. La suficiencia muestral se determinó mediante criterio de saturación teórica, considerándose alcanzada cuando los grupos focales dejaron de aportar categorías conceptuales sustancialmente nuevas. Cada grupo estuvo formado por cinco a siete profesionales, en línea con las recomendaciones metodológicas para asegurar la diversidad de opiniones sin comprometer la profundidad del intercambio^15^. Las sesiones fueron moderadas por la investigadora principal y contaron con una observadora no participante encargada de registrar aspectos no verbales y dinámicas relacionales, reforzando así la fiabilidad del proceso de recolección de datos.

### Recolección de datos

La recolección de datos se llevó a cabo mediante la técnica cualitativa de grupos focales con una guía semiestructurada de preguntas. Esta metodología fue seleccionada por su capacidad para explorar percepciones, significados y experiencias compartidas entre profesionales que comparten un mismo contexto asistencial. Permite generar información rica y contrastada a través de la interacción grupal, el intercambio de experiencias y la construcción colectiva de sentido, elementos especialmente relevantes cuando se analiza la experiencia del paciente y los resultados en salud reportados por el mismo.

Las discusiones siguieron una guía semiestructurada previamente elaborada, que incluyó preguntas centradas en cinco áreas: 1) percepción general de la experiencia del paciente; 2) factores determinantes de la experiencia del paciente; 3) indicadores observables relacionados con los resultados en salud reportados por el paciente; 4) oportunidades de mejora en los procesos asistenciales; y 5) modos de integrar PREMs y PROMs en la mejora continua de la práctica enfermera (Anexo I).

Las sesiones, realizadas en espacios reservados dentro de los propios hospitales, fueron transcritas de forma automática en tiempo real mediante Word dictado, previa obtención del consentimiento informado. Para ello, se dispuso un ordenador con micrófono en la sala donde se desarrollaron los grupos focales, permitiendo la conversión simultánea de las intervenciones orales a texto escrito. No fueron realizadas grabaciones de audio o vídeo. Posteriormente los textos fueron revisados para detectar errores evidentes de transcripción. Se garantizó el anonimato mediante la codificación de participantes y la eliminación de información identificativa. Los grupos focales tuvieron una duración mínima de 50 minutos y máxima de 90 minutos.

La investigadora principal, enfermera con formación específica en investigación y experiencia previa en conducción de grupos focales, actuó como moderadora en todas las sesiones. La observadora no participante contaba igualmente con experiencia investigadora en análisis cualitativo. No existía relación jerárquica directa entre las investigadoras y las participantes, aunque compartían pertenencia institucional. Para minimizar sesgos derivados de supuestos previos, se empleó una guía semiestructurada común y se promovió una actitud reflexiva durante todo el proceso analítico mediante contraste continuo entre investigadoras.

Este proyecto recibió la aprobación ética del Comité de Ética de Investigación de la Universidad Camilo José Cela (código 25_25_PREOS). Todos los participantes proporcionaron su consentimiento informado por escrito antes de participar. Asimismo, consintieron verbalmente la transcripción simultánea. El estudio se adhirió a la Guía *Standards for Reporting Qualitative Research* (SRQR)^16^ y a lista de verificación *COnsolidated criteria for REporting Qualitative research* (COREQ)^17^ para el informe de investigación cualitativa.

### Análisis de datos

El análisis se desarrolló siguiendo un enfoque de análisis temático inductivo, conforme a las recomendaciones metodológicas para estudios cualitativos descritas en Ritchie y Lewis^15^. Este proceso se estructuró en cuatro fases:

- Familiarización: dos investigadoras realizaron de forma independiente varias lecturas completas de las transcripciones para obtener una visión preliminar y detectar temas emergentes.
- Codificación: se desarrolló un sistema inicial de códigos combinando códigos descriptivos y analíticos mediante consenso.
- Categorización: los códigos se agruparon en categorías superiores, generando unidades temáticas coherentes que permitieron organizar los hallazgos. Este proceso permitió construir un árbol de codificación progresivo organizado en temas y subtemas.
- Síntesis interpretativa: se sintetizaron los resultados mediante la identificación de patrones transversales y relaciones entre temas, integrando citas textuales representativas como evidencia.

Las citas textuales incluidas en los resultados fueron sometidas a una edición mínima orientada a mejorar su legibilidad, eliminando artefactos derivados de la transcripción automática, muletillas o interrupciones no relevantes para la interpretación, sin modificar el significado original de los discursos.

La codificación y organización de los datos se realizó de forma manual utilizando comparación iterativa entre transcripciones. No se empleó un software específico para este análisis cualitativo. La saturación teórica se consideró alcanzada cuando no emergieron nuevas categorías conceptuales relevantes.

Para asegurar la calidad y rigor metodológico, se aplicaron criterios de credibilidad, dependencia y confirmabilidad mediante triangulación entre investigadoras, contraste interno de categorías, registro detallado del proceso analítico, y uso de citas literales para sostener la interpretación. No obstante, no se realizó devolución individual de transcripciones ni validación formal de los hallazgos por parte de los participantes, aspecto reconocido como limitación metodológica.

## RESULTADOS

No se registraron rechazos ni abandonos una vez aceptada la participación. Se llevaron a cabo cinco grupos focales con un total de 32 enfermeras. La gran mayoría de las participantes fueron del sexo femenino y predominaron las que trabajaban en hospitalización (Tabla 1).

**Tabla 1:** Datos demográficos de las participantes (n=32)

Características	n
Unidad	
Urgencias	5
Planta de hospitalización	12
Quirófano	1
UCI adultos	6
UCI neonatal	4
Oncología	4
Sexo	
Mujer	26
Hombre	6

UCI: unidad de cuidados intensivos.

Como resultado del análisis se obtuvieron seis unidades temáticas: comunicación y vínculo terapéutico; cuidado integral (confort, seguridad y dolor); información y educación sanitaria; evaluación de PREMs y PROMs; limitaciones organizativas; e innovación e investigación orientadas al paciente, que posteriormente fueron integradas en una matriz temática completa que sintetiza los hallazgos del estudio (Tabla 2).

**Tabla 2:** Resultados del análisis temático

Subtemas	Códigos
*Unidad temática 1. Comunicación, vínculo y trato humano*	
1.1. Relación terapéutica	- Escucha activa
	- Acompañamiento y apoyo emocional
	- Generación de confianza
	- Presencia continua
1.2 Presentación y trato personalizado	- Presentación por nombre y rol
	- Uso de nombre preferido del paciente
	- Coherencia del personal
*Unidad temática 2. Cuidado integral: confort, seguridad y alivio del dolor*	
2.1. Confort físico	- Intimidad
	- Temperatura
	- Descanso
	- Comodidad en espacios
	- Iluminación
2.2. Seguridad clínica	- Prevención de infecciones
	- Accesos vasculares adecuados y permeabilidad
	- Prevención de úlceras
	- Aplicación de protocolos de seguridad
2.3. Manejo del dolor	- Dolor como indicador central de bienestar
	- Dolor como resultado relevante reportado por el paciente
*Unidad temática 3. Información, comunicación clínica y educación sanitaria*	
3.1. Información durante el proceso	- Explicación de pruebas y procedimientos
	- Mediación entre paciente y médico
	- Adaptación de la información
	- Aumentar la comprensión
3.2. Educación al alta	- Entrega de materiales escritos que favorezcan la adherencia
	- Reeducación de hábitos
	- Reforzar autocuidados
	- Prevención de complicaciones
3.3. Coordinación en comunicación	- Trabajo en equipo
	- Habilidades comunicativas del equipo
*Unidad temática 4. Evaluación sistemática de PREMs y PROMs*	
4.1. Recogida de información sobre experiencia del paciente	- Encuestas de experiencia del paciente
	- Sistemas digitales que alertan sobre la insatisfacción del usuario
	- Supervisión
	- Conversaciones informales
4.2. Resultados relevantes para el paciente	- Dolor
	- Movilidad
	- Descanso
	- Evolución percibida del estado de salud
*Unidad temática 5. Limitaciones organizativas y estructurales*	
5.1. Falta de tiempo y carga asistencial	- Falta de tiempo
	- Elevada carga
	- Excesivo tiempo dedicado a registros limita el cuidado
5.2. Infraestructura insuficiente	- Agrandar espacios
	- Infraestructura para familias
*Unidad temática 6. Innovación, humanización e investigación orientadas al paciente*	
6.1. Nuevos proyectos de humanización	- Sacar al paciente a exteriores (UCI adultos)
	- Material informativo
	- Música, objetos personales y orientación
	- Salas familiares adecuadas
6.2. Innovación tecnológica	- Canal informativo del paciente
	- Información sobre tiempos de espera
	- Tarjeta inteligente
	- Dispositivos de monitorización con IA
6.3. Práctica avanzada y Coordinación interdisciplinar	- Despliegue de EPAs
	- Comunicación con equipo
	- Monitorización y algoritmos para el dolor
	- Continuidad asistencial

### Tema 1. Comunicación, vínculo y trato humano

Este tema emerge como el principal determinante de la experiencia del paciente, siendo transversal a todas las unidades. Las enfermeras describen que la experiencia del paciente y la familia está profundamente condicionada por la calidad de la relación terapéutica, el trato personalizado y la percepción de presencia y disponibilidad enfermera, incluso por encima de otros cuidados técnicos.

Las participantes identifican la capacidad de la enfermera para establecer una relación basada en la escucha activa, el acompañamiento y apoyo emocional, la generación de confianza y la presencia continuada. Estos elementos se describen como esenciales para modular la experiencia del paciente y favorecer una vivencia positiva del proceso asistencial especialmente relevante en contextos de hospitalización prolongada, unidad de cuidados intensivos y oncología, así como en contextos pediátricos y neonatales, donde la familia se convierte también en receptora directa del cuidado.

*Al final podemos mantenerles informados y cuidarles bien. La parte técnica se da por hecha; lo que añadimos es, sobre todo, la comunicación y el trato para que se sientan mejor.* (Enfermera, planta de hospitalización)

Por otro lado, identifican las prácticas relacionadas con la presentación por nombre y rol profesional, el reconocimiento del paciente como persona única al hacer uso del nombre preferido del paciente y la coherencia del personal asistencial como cruciales para la modulación de la experiencia del paciente. Las enfermeras señalan que estas acciones contribuyen a personalizar el cuidado, reducir la ansiedad y reforzar la continuidad percibida del proceso asistencial.

*Los pacientes lo agradecen mucho, porque no es lo mismo que alguien con una cara conocida se presente al despertar y te diga: “Soy Belén y voy a estar contigo dentro del quirófano; cualquier cosa que necesites, me lo dices”. Luego, cuando entras en quirófano, vuelves a ver a Belén, que es quien te ha saludado antes, y creo que eso lo agradecen mucho.* (Enfermera, quirófano)

### Tema 2. Cuidado integral: confort, seguridad y alivio del dolor

Esta unidad temática recoge los aspectos del cuidado orientados al bienestar físico, la seguridad clínica y el alivio del dolor. Las participantes destacan que el cuidado integral constituye un eje central tanto de la experiencia del paciente como de los resultados en salud, y que estos elementos deben ser considerados indicadores clave de calidad asistencial. Son tres los elementos centrales que marcan la diferencia en el cuidado integral y que repercuten en una mejor experiencia del paciente. En primer lugar, el confort físico entendido como las condiciones que favorecen el bienestar del paciente, incluyendo la intimidad, la temperatura, el descanso, la comodidad de los espacios y la iluminación. Las enfermeras identifican el entorno físico como un factor que condiciona la experiencia del paciente y de las familias.

*Luego el frío, que no tengan siempre frío ya no solamente a nivel físico, sino que a nivel confort que estén lo más cómodos posible.* (Enfermera, planta de hospitalización)

Por otro lado, La seguridad se describe como un componente intrínseco del cuidado enfermero y un elemento que las participantes consideran estrechamente relacionado con la experiencia del paciente y con determinados resultados en salud percibidos por este.

*Otro aspecto relacionado con la seguridad es que muchos familiares y pacientes comentan: “Si estuviera en la UCI, allí me cuidarían mejor”. Eso es porque tiene que entender también que aquí son dos pacientes por una enfermera. Que son cuidados totalmente diferentes y que las enfermeras permanecen con un paciente 2 o 3 horas sin parar y que aunque sean 3 pacientes, que todo paciente que viene se le mira cómo está con muchos cuidados.* (Enfermera, UCI adultos)

*Todos los elementos que tengan que ver con seguridad del paciente, de riesgo de caída, riesgos de úlcera, errores de medicación e identificación inequívoca.* (Enfermera, planta de hospitalización)

Por último, el dolor es identificado como indicador central de bienestar. Las enfermeras coinciden en señalar el dolor como uno de los principales resultados en salud reportados por los pacientes y un indicador especialmente relevante para evaluar su bienestar y evolución clínica.

*Creo que lo primero que siempre hay que intentar es que los pacientes estén sin dolor para cualquier técnica. También por ejemplo ahora hay que sacarle una analítica pues siempre que esté con analgesia o le damos suero con sacarosa para que esté cómodo.* (Enfermera, UCI neonatal)

### Tema 3. Información, comunicación clínica y educación sanitaria

Las enfermeras refieren que la experiencia del paciente también versa sobre aspectos relacionados con la transmisión de información clínica y la educación sanitaria a lo largo del proceso asistencial. Las enfermeras destacan su papel como referentes informativos del paciente, especialmente en contextos de alta complejidad. Además, refleja el papel clave de la enfermería como mediadora de la información clínica, especialmente cuando existen desajustes comunicativos con otros profesionales. Según las participantes, los pacientes se sienten mejor cuando ellas se implican en la explicación de pruebas y procedimientos, la adaptación del lenguaje y la mediación entre el paciente y otros profesionales. La función de la enfermera se orienta a mejorar la comprensión del proceso asistencial y reducir la incertidumbre, favoreciendo una mejor experiencia asistencial y pudiendo contribuir a resultados percibidos más favorables.

*La información que se le da al paciente para que sepa qué va a pasar y cuál es el siguiente paso también ayuda a que avance. Por ejemplo, explicarle cuándo va a empezar a caminar y cómo va a hacerlo. Es importante que no tenga esa incertidumbre de preguntarse cuándo podrá hacerlo, porque muchas veces la información no les llega de forma clara.* (Enfermera, planta de hospitalización)

Las intervenciones educativas orientadas a reforzar los autocuidados, la adherencia terapéutica, la prevención de complicaciones tras el alta y la educación al alta se identifican como un elemento clave para la continuidad asistencial y la mejora de resultados en salud percibidos por el paciente y la continuidad de los cuidados tras el alta.

*Recientemente hemos empezado a utilizar las recomendaciones al alta que se le entrega al paciente en un informe sobre la administración de la heparina subcutánea, hemos visto que eso funciona. El paciente se va a casa mejor informado y mejora sus resultados a largo plazo.* (Enfermera, planta de hospitalización)

Por último, recoge la importancia del trabajo en equipo y de las habilidades comunicativas compartidas. Las enfermeras señalan que una comunicación coordinada entre profesionales favorece la coherencia de la información transmitida al paciente.

*Muchos pacientes atribuyen sus molestias y quejas a problemas de comunicación con el médico. Dicen: “El médico me ha dicho esto, pero se le ha olvidado comunicártelo”, o “tú no puedes hacer nada porque no te lo han pautado”. Eso les genera desconfianza y les molesta mucho.* (Enfermera, planta de hospitalización)

### Tema 4. Evaluación sistemática de PREMs y PROMs

Esta unidad temática describe la percepción de las enfermeras sobre la recogida y utilización de medidas de experiencia y resultados reportados por los pacientes. Se reconoce el valor de estas herramientas para la mejora de la calidad, aunque se identifican áreas de desarrollo en su sistematización. Incluye los métodos utilizados para recoger la experiencia del paciente, como encuestas, sistemas digitales, supervisión y conversaciones informales. Las participantes señalan la necesidad de integrar estos datos de forma más estructurada en la gestión y mejora de los cuidados.

*Cuando hablo con el paciente, no solo le pregunto cómo le ha parecido la información que le hemos dado; también aprovecho para recoger toda la información que puedo, y no únicamente durante las visitas.* (Enfermero, planta de hospitalización)

Las enfermeras identifican dimensiones como el dolor, la movilidad, el descanso y la evolución percibida del estado de salud como resultados especialmente relevantes para los pacientes. Este hallazgo refleja cómo las participantes reconocen estos aspectos como indicadores significativos del bienestar y la recuperación, aunque su evaluación en la práctica clínica puede realizarse mediante diferentes fuentes de información, incluyendo medidas reportadas por los propios pacientes y registros profesionales. Asimismo, se pone de manifiesto la necesidad de incorporar de forma más sistemática la perspectiva del paciente en la valoración de estos resultados.

Algunas participantes también relacionaron la evaluación de estos resultados con indicadores clínicos y registros asistenciales utilizados habitualmente en la práctica diaria.

*Los cuidados que se están registrando pueden exportarse y cuantificarse. Al final, se trata de medir aspectos como la tensión arterial, la rapidez de respuesta o la realización de las escalas.* (Enfermera, planta de hospitalización)

### Tema 5. Limitaciones organizativas y estructurales

Esta unidad temática recoge los factores organizativos y estructurales que dificultan la provisión de cuidados centrados en la persona y la implementación sistemática de mejoras basadas en PREMs y PROMs. Se refiere a la presión asistencial y a la sobrecarga de trabajo, que limitan el tiempo disponible para la atención directa y pueden comprometer la calidad percibida de los cuidados.

*Yo creo que el factor más importante es el tiempo porque el tiempo es lo que hace que haya sobrecarga de trabajo y poder acoger al paciente de una manera más sencilla.* (Enfermera, planta de hospitalización)

*Siento que no me da la vida. Muchas veces los propios pacientes me dicen: “No has parado; te veo corriendo todo el tiempo por el pasillo”.* (Enfermera, planta de hospitalización)

Incluye las limitaciones relacionadas con los espacios físicos y los recursos disponibles para pacientes y familias, identificadas como un factor que influye negativamente en la experiencia asistencial.

*Es un tema de optimización de espacios, necesitaríamos más sillones para las medicaciones intravenosas. Estaría bien ampliar la urgencia.* (Enfermera, urgencias)

### Tema 6. Innovación, humanización e investigación orientadas al paciente

Esta unidad temática agrupa las propuestas y experiencias de mejora identificadas por las enfermeras, orientadas a generar propuestas de mejora para la organización de los cuidados y la experiencia del paciente. Incluye propuestas relacionadas con el desarrollo de roles de práctica avanzada, la coordinación interdisciplinar y la continuidad asistencial, orientadas a mejorar la calidad y personalización de los cuidados.

*Hay que investigar en humanizar más las UCIs, aspectos como dejar a la familia que esté más tiempo, que participen en los cuidados y evaluarlo.* (Enfermera, UCI adultos)

Hace referencia a la incorporación de herramientas tecnológicas para mejorar la información al paciente, optimizar los tiempos de espera y facilitar la monitorización de resultados en salud.

*Desde hace tiempo tengo la idea de crear un canal de información para el paciente ingresado. Tenemos televisores en cada habitación y podría existir un canal cerrado donde el paciente pudiera consultar quién es su médico responsable, cuál es su dieta, qué pruebas tiene programadas o quién es la enfermera de guardia. Esa información podría ayudarle a reducir la incertidumbre. Que encienda la televisión y piense: “Vale, mañana me toca esta prueba”.* (Enfermero, planta de hospitalización)

Incluye el desarrollo de roles de práctica avanzada, equipos especializados y el uso de protocolos y algoritmos orientados a mejorar el control del dolor, la continuidad asistencial y los resultados reportados por los pacientes.

*… la enfermería de práctica avanzada en pacientes con curas y cuidados a domicilio o seguimientos al alta de pacientes de UCI.* (Enfermera, UCI adultos)

## DISCUSIÓN

Los resultados de este estudio ponen de manifiesto que la comunicación, el trato humano y el establecimiento de un vínculo terapéutico emergen como los principales determinantes de la experiencia del paciente, incluso por encima de los cuidados técnicos, según las enfermeras. Este hallazgo refuerza el modelo de atención centrada en la persona y sitúa a la enfermería como actor nuclear en la construcción de la experiencia asistencial.

La literatura internacional respalda de forma consistente la relación entre comunicación efectiva, confianza y experiencia positiva del paciente. Una comunicación clara, empática y continuada se asocia con una experiencia más satisfactoria y mayor percepción de calidad, independientemente del resultado clínico final^18^^,^^19^. Desde la perspectiva de enfermería, la comunicación no se percibe como un complemento del cuidado, sino como un componente inseparable de la práctica profesional. Este hallazgo coincide con investigaciones cualitativas previas que describen el vínculo terapéutico como un elemento central del cuidado enfermero^9^.

Un aspecto especialmente relevante es que las herramientas actuales para medir la experiencia del paciente suelen centrarse en dimensiones comunicativas como la información recibida, la claridad o el trato recibido^20^. Sin embargo, captan de forma limitada la profundidad del vínculo terapéutico descrito por las enfermeras en este estudio. Esto sugiere la necesidad de evolucionar hacia PREMs sensibles a la intervención enfermera que integren dimensiones relacionales más complejas desarrollando instrumentos más refinados para evaluar la experiencia desde una perspectiva de cuidado.

El confort, la seguridad clínica y, especialmente, el manejo del dolor, emergen en este estudio como elementos relacionados con PREMs y PROMs. Las enfermeras identifican el dolor como un indicador prioritario de bienestar, coincidiendo con estudios previos^21^. La prominencia del dolor en los discursos enfermeros puede explicarse, en parte, porque se trata de uno de los resultados más sistemáticamente evaluados en la práctica clínica, lo que refleja su alto grado de institucionalización. Estudios previos han demostrado que los PROMs que se integran de forma rutinaria en los sistemas asistenciales son apropiados más fácilmente por los profesionales y utilizados para guiar el cuidado diario^3^.

Asimismo, las participantes destacan la influencia del entorno físico, la intimidad y la prevención de complicaciones en la experiencia y en los resultados funcionales y emocionales del paciente. Estos hallazgos refuerzan la idea de que el cuidado integral actúa como un puente conceptual entre PREMs y PROMs, apoyando modelos que abogan por una evaluación integrada de experiencia y resultados desde una perspectiva de cuidados^1^.

Este estudio confirma el papel clave de la enfermería como mediadora de la información clínica y referente educativo del paciente a lo largo del proceso asistencial. La educación sanitaria y la información al alta son percibidas como intervenciones con impacto más allá del ingreso hospitalario, influyendo en la adherencia terapéutica, los autocuidados y la evolución clínica posterior. La evidencia disponible respalda que las intervenciones educativas lideradas por enfermería se asocian con mejores resultados a medio y largo plazo, incluyendo reducción de reingresos y mejora de la calidad de vida reportada por el paciente^12^. Estos hallazgos permiten introducir la continuidad asistencial como un resultado sensible a la práctica enfermera, y refuerzan la necesidad de integrar PROMs post-alta en la evaluación de procesos asistenciales liderados por enfermería.

Las enfermeras también identifican déficits de comunicación interprofesional como una fuente relevante de mala experiencia del paciente, coincidiendo con estudios que señalan la incoherencia informativa como uno de los principales factores de insatisfacción y desconfianza^2^.

A pesar del alto reconocimiento del valor de las experiencias y resultados reportados por el paciente, este estudio identifica una brecha significativa entre su potencial teórico y su uso real en la práctica clínica, ya que predomina una recogida informal de información frente a sistemas estructurados y sistemáticos. Esta paradoja ha sido descrita previamente en otros contextos sanitarios. Kynoch y col^10^ identificaron que, aunque la recogida de PREMs y PROMs está ampliamente extendida, existe una utilización limitada de estos datos para impulsar cambios en la práctica clínica y en los servicios, señalando además barreras relacionadas con la integración organizativa, la capacidad de interpretación de los resultados y la disponibilidad de recursos para implementar mejoras. La falta de tiempo, la sobrecarga asistencial y las limitaciones estructurales emergen como amenazas directas para PREMs y PROMs. Las enfermeras describen un conflicto constante entre las exigencias administrativas y el tiempo disponible para el cuidado directo. Autores como Hansen y col ^22^ identificaron que las experiencias de las enfermeras con las PROMs dependían del tiempo disponible y de la estrategia de su implementación, lo que limitaba su capacidad para utilizarlas, a pesar de que eran considerados potencialmente importantes para apoyar la práctica. Estos hallazgos son consistentes con la revisión sistemática de Bonsel y col ^23^, que señala que la utilización de PROMs resulta más efectiva cuando la información obtenida se integra en procesos de monitorización, toma de decisiones y mejora asistencial, mientras que la evidencia sobre su aplicación organizativa sigue siendo limitada. En este sentido, comprender cómo los profesionales de enfermería interpretan y valoran estas medidas puede contribuir a identificar estrategias factibles para su incorporación en la práctica clínica y en iniciativas de mejora de la calidad.

El carácter propositivo del discurso enfermero constituye uno de los hallazgos más relevantes del estudio. La emergencia de iniciativas de humanización, innovación tecnológica y práctica avanzada refleja el potencial de la investigación cualitativa como generadora de cambio. Los hallazgos obtenidos ofrecen una base conceptual útil para orientar futuras iniciativas de mejora asistencial, innovación organizativa e investigación aplicada relacionadas con la integración de PREMs y PROMs en la práctica enfermera^6^. Desde una perspectiva de gestión, los resultados sugieren la conveniencia de desarrollar sistemas estructurados para la recogida y utilización de información reportada por los pacientes, reforzar las estrategias de educación sanitaria y continuidad asistencial, e incorporar indicadores relacionados con la experiencia del paciente en los procesos de evaluación y mejora de la calidad^10^.

Resulta especialmente relevante que las propuestas de innovación tecnológica y humanización no aparezcan como dimensiones contrapuestas, sino complementarias. Las participantes identifican herramientas tecnológicas orientadas a mejorar la información, la comunicación y la continuidad asistencial, al tiempo que proponen intervenciones centradas en el bienestar emocional y la personalización de los cuidados. Estos hallazgos son coherentes con enfoques contemporáneos de atención centrada en la persona, que plantean que la tecnología puede actuar como facilitadora de experiencias más humanizadas cuando se orienta a responder a las necesidades y preferencias de los pacientes^24^.

Un hallazgo relevante es que las participantes tienden a integrar en un mismo marco conceptual elementos procedentes de diferentes sistemas de evaluación de la calidad asistencial. Aspectos como la seguridad clínica, la prevención de complicaciones o determinadas escalas asistenciales fueron mencionados junto a dimensiones más próximas a los PREMs y PROMs. Esta observación no debe interpretarse como una equivalencia conceptual entre dichos constructos, sino como reflejo de la visión integral que las enfermeras tienen sobre la calidad de los cuidados y los factores que influyen en la experiencia y los resultados percibidos por los pacientes.

Este estudio constituye una fase exploratoria destinada a comprender las percepciones profesionales sobre la utilidad de los PREMs y PROMs y a identificar áreas prioritarias de mejora. Futuras investigaciones podrán utilizar estos resultados como base para diseñar, implementar y evaluar intervenciones dirigidas a optimizar los cuidados, fortalecer la atención centrada en la persona y analizar el impacto de la integración sistemática de PREMs y PROMs en los resultados asistenciales.

Desde una perspectiva de transferibilidad, aunque este estudio se desarrolló en un contexto hospitalario privado, los retos identificados en torno a la integración sistemática de PREMs y PROMs, la presión asistencial, la necesidad de fortalecer la comunicación clínica y la orientación hacia cuidados centrados en la persona son compartidos por múltiples organizaciones sanitarias. En este sentido, los hallazgos pueden contribuir como marco interpretativo para futuras iniciativas en otros entornos asistenciales, siempre que su implementación se adapte a las particularidades estructurales y organizativas de cada sistema sanitario. Asimismo, futuras investigaciones deberían incorporar la perspectiva de pacientes y familiares para contrastar y complementar las percepciones profesionales identificadas en este estudio

Entre las fortalezas de este estudio destacan su diseño multicéntrico, la inclusión de unidades asistenciales con distintos niveles de complejidad, el rigor metodológico y el uso sistemático del análisis temático. Entre sus limitaciones, debe señalarse que los hallazgos proceden de un grupo hospitalario privado con características organizativas específicas, lo que condiciona su transferibilidad directa a otros entornos asistenciales. La realización de grupos focales en el propio entorno laboral podría haber favorecido sesgos de deseabilidad social o influido en la expresión de opiniones potencialmente críticas hacia la organización. Asimismo, el análisis recoge exclusivamente la perspectiva enfermera, sin incorporar la visión de pacientes, familiares ni otros profesionales sanitarios. Por último, no se realizó devolución de transcripciones o validación formal de resultados con las participantes, lo que puede limitar la validez externa de la interpretación realizada. Asimismo, la ausencia de grabaciones de audio limitó la posibilidad de realizar una verificación posterior completa de las transcripciones, si bien se adoptaron medidas de revisión y contraste para minimizar posibles errores de registro. Por último, la representación de algunas áreas asistenciales fue desigual. En particular, la participación de una única enfermera de quirófano limita la posibilidad de interpretar de forma específica las percepciones de este ámbito asistencial, por lo que los resultados deben entenderse desde una perspectiva global y no comparativa entre unidades.

No obstante, los temas identificados reflejan dimensiones nucleares de la práctica enfermera y de la gestión de la calidad, descritas también en otros contextos sanitarios. Su aplicabilidad a sistemas públicos o a modelos organizativos diferentes debe interpretarse con cautela y considerando las particularidades estructurales, de recursos y de cultura organizativa de cada institución.

En conclusión, las enfermeras participantes perciben los PREMs y PROMs como herramientas útiles para comprender la experiencia y los resultados reportados por los pacientes, así como para identificar oportunidades de mejora en la calidad de los cuidados. La comunicación, el trato humano y el vínculo terapéutico emergen como los principales determinantes de la experiencia del paciente, situando a la enfermería como actor clave en la humanización de los cuidados y en la construcción de valor asistencial.

El estudio evidencia el papel estratégico de la enfermería en la información clínica y la educación sanitaria, especialmente en la transición al alta, lo que refuerza la necesidad de incorporar indicadores de continuidad asistencial y PROMs post-alta en los modelos de evaluación de procesos enfermeros. No obstante, las limitaciones organizativas, la sobrecarga asistencial y la falta de sistemas estructurados dificultan el uso sistemático de estas medidas, señalando áreas críticas de mejora para la gestión. Los resultados obtenidos aportan una mejor comprensión de cómo las enfermeras interpretan el potencial de los PREMs y PROMs en su práctica profesional y pueden contribuir a orientar futuras iniciativas de mejora e investigación dirigidas a fortalecer modelos de atención centrados en la persona.

## Data Availability

Los datos pueden consultarse a través de la autora de correspondencia.

## ANEXO I

### Guía semiestructurada para los grupos focales

1. Percepción general de la experiencia del paciente: ¿Cómo describiríais la influencia de los cuidados de enfermería en la experiencia que los pacientes tienen durante su proceso asistencial?
2. Factores determinantes de la experiencia del paciente (PREMs) Desde vuestra perspectiva, ¿qué aspectos de la atención enfermera impactan más en la experiencia y satisfacción de los pacientes?
3. Estrategias de mejora de la experiencia del paciente ¿Qué medidas o prácticas consideráis más efectivas para mejorar la experiencia del paciente en vuestra unidad?
4. Resultados reportados por el paciente (PROMs) ¿Qué indicadores observáis en vuestra práctica diaria que reflejen mejor la evolución, el bienestar o el estado de salud de los pacientes?
5. Procesos asistenciales y organización del cuidado ¿Qué cambios o mejoras en la organización o en los procesos de trabajo podrían favorecer una mejor experiencia y mejores resultados en salud del paciente?
6. Integración de la voz del paciente en la mejora continua ¿De qué manera podría la enfermería incorporar de forma más sistemática la información obtenida de los PREMs y PROMs en la mejora continua de los cuidados.
